# MicroRNAs: Diverse Mechanisms of Action and Their Potential Applications as Cancer Epi-Therapeutics

**DOI:** 10.3390/biom10091285

**Published:** 2020-09-07

**Authors:** Anna Sadakierska-Chudy

**Affiliations:** Department of Genetics, Faculty of Medicine and Health Sciences, Andrzej Frycz Modrzewski Krakow University, 30-705 Krakow, Poland; asadakierska-chudy@afm.edu.pl

**Keywords:** Ago, DNMT inhibitor, epi-miRs, HDAC, lncRNA, virial vector

## Abstract

Usually, miRNAs function post-transcriptionally, by base-pairing with the 3′UTR of target mRNAs, repressing protein synthesis in the cytoplasm. Furthermore, other regions including gene promoters, as well as coding and 5′UTR regions of mRNAs are able to interact with miRNAs. In recent years, miRNAs have emerged as important regulators of both translational and transcriptional programs. The expression of miRNA genes, similar to protein-coding genes, can be epigenetically regulated, in turn miRNA molecules (named epi-miRs) are able to regulate epigenetic enzymatic machinery. The most recent line of evidence indicates that miRNAs can influence physiological processes, such as embryonic development, cell proliferation, differentiation, and apoptosis as well as pathological processes (e.g., tumorigenesis) through epigenetic mechanisms. Some tumor types show repression of tumor-suppressor epi-miRs resulting in cancer progression and metastasis, hence these molecules have become novel therapeutic targets in the last few years. This review provides information about miRNAs involvement in the various levels of transcription and translation regulation, as well as discusses therapeutic potential of tumor-suppressor epi-miRs used in in vitro and in vivo anti-cancer therapy.

## 1. Introduction

Although research into RNA biology has been ongoing for more than two decades, almost each year brings new discoveries. Until recently, it was thought that microRNAs (miRNAs) act mainly in the cytoplasm at the post-transcriptional level. Interestingly, miRNAs can exert regulatory effect both in the cell (i.e., cytoplasm and nucleus) in which they are produced and in neighboring cells. The latter intracellular transfer of miRNA is mediated by gap junction channels or exosomes [1]. Interestingly, mature miRNAs can regulate one or more mRNA targets, but also a single mRNA transcript can be bound and regulated by many different miRNAs. It is estimated that each miRNA can recognize ~100–200 target sites of the transcriptome and the inhibitory effect on expression can be achieved at 1000 copies per cell [1,2]. miRNAs can recognize and bind to 3′UTR, 5′UTR and coding sequence of their targets’ mRNA, as well as to promoter regions. Considering miRNAs variety and localization, cell type and cell state, their possibilities to regulate gene expression are limitless.

## 2. Inhibition or Activation of Translation

Mature miRNAs (mainly guide strands) form a complex with Argonaut (AGO) proteins called miRNA-induced silencing complex (miRISC) which interact with other proteins including DICER, TRBP, PACT and GW182. The miRNA specific region called ‘seed sequence’ (which includes nucleotides between 2 and 8, counting from the 5′ end of the miRNA) base-pairs with miRNA recognition elements (MREs) located on mRNA targets. miRISC complex binding to 3′UTR leads to mRNA cleavage or mRNA decay and finally translation inhibition. For example, full complementarity of the seed region to MRE induces AGO-2 endonuclease activity resulting in mRNA cleavage and destabilization of AGO2-miRNA interaction that ultimately promotes miRNA degradation [3,4]. In contrast, incomplete complementarity prevents AGO-2 endonuclease activity but initiates the recruitment of GW182 protein family leading to mRNA decay. GW182 interacts with PABPC (polyadenylate-binding protein) that promotes efficient mRNA deadenylation by recruiting poly(A)-deadenylase complexes (PAN2-PAN3 and CCR4-NOT). Subsequently, mRNA-decapping enzymes (DCP1-DCP2) recognize and remove the 5′-cap from mRNA transcripts making mRNA susceptible to degradation by 5′-3′ exoribonuclease 1 (XRN1) [5,6]. A recent study has revealed that mRNA decay is responsible for a large majority (66–90%) of miRNA-mediated translation repression [7]. Moreover, miRISC can inhibit translation at the initiation step probably through dissociation of eukaryotic initiation factors 4A (eIFA4-I and eIFA4-II) making it difficult for ribosome scanning and assembly of the eIF4F translation initiation complex [8,9].

In addition, miRNAs can also bind to target sites in the coding region and inhibit translation. Probably, target sequences in the coding regions are used when the 3′UTR are too short or adjust to protein abundance of alternative splice variants [10]. For example, Cardinali et al. have identified that the *AHNAK* gene contains a *miR-222* target sequence within the coding region hence *miR-222* can directly down-regulate its expression [11]. Additionally, a study by Splengler et al. has revealed an abundance of miRNA target sites in gene coding regions [12].

Surprisingly, some studies have reported that miRNAs binding to 3′UTR or 5′UTR regions can up-regulate gene expression by increasing translation rates of proteins.

It was demonstrated that translation activation can depend on the cell cycle state and proteins that are bound to the AGO2-miRNA complex within the 3′UTR. Indeed, in serum starved cells Fragile-X-mental retardation related protein 1 (FXR1) was recruited by the AGO2-miRNA complex associated with AU-rich elements (AREs) at the 3′UTR and activated translation [13]. The miRNA-mediated activation of transcription has been observed also in other quiescent cells, such as immature oocytes of the *Xenopus* (e.g., *Myt1* mRNA via AGO-miR16-FXR1 complex) [14].

Furthermore, it was revealed that miRNAs that bind to the 5′UTR can enhance translation through various mechanisms. For example, liver specific *miR-122* binding to RNA of hepatitis C virus protects the single-stranded 5′ end from cytosolic exonuclease activities (increasing RNA stability against Xrn1) and enhances the recruitment of ribosomes as well as possibly providing a scaffold for binding of other essential factors for translation [15,16,17,18]. Another example is *miR-10a* that interacts with the 5′ terminal oligopyrimidine (5′TOP) motif of ribosomal protein mRNAs and enhance their translation during amino acid starvation [19]. Moreover, *miR-346*, produced mostly in brain tissues, binds to 5′UTR of the receptor interacting protein 140 (RIP140) mRNA facilitating its association with the polysomes and finally activating translation via an AGO2-independent manner [20].

## 3. Suppression or Enhancement of Transcription

Surprisingly, studies have shown bidirectional transport of the core components of miRNA silencing machinery. It was demonstrated that several proteins including Importin-8, Importin α/β (Impα/Impβ) and Exportin-1 (XPO1) mediate shuttling of key RISC components from the cytoplasm to the nucleus, moreover, XPO1 is able to transport the nuclear RISC (miRNA-Ago-TNRC6A complex) to the cytoplasm [21,22].

**Figure 1:**
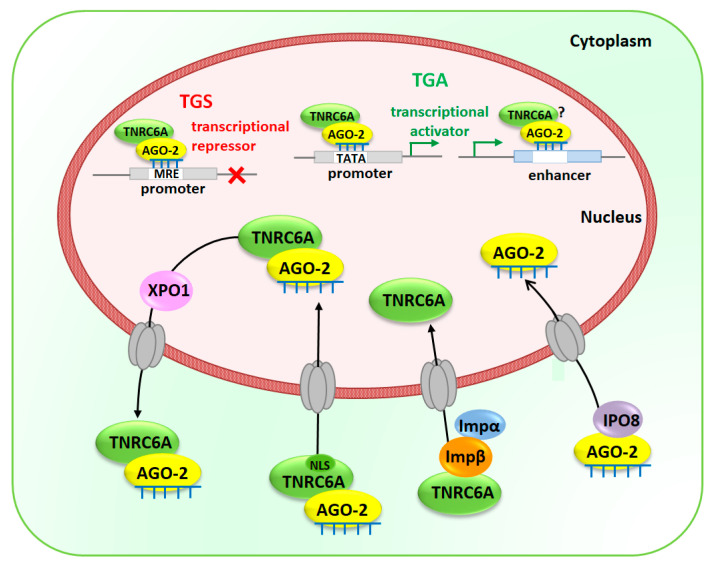
Transport of mature miRNAs and components of RISC (RNA-induced silencing complex) into the nucleus. TNRC6A is shuttled from the cytoplasm into the nucleus either via its own NLS sequence when it interacts with miRNA-AGO complex or independently via its interaction with Importin β (Imp β) and Importin α (Impα). While mature miRNAs loaded into AGO-2 are translocated into the nucleus by Importin 8 (IPO8) miRNA-AGO-TNRC6A complex can be exported back to the cytoplasm by Exportin 1 (XPO1). In the nucleus, miRISC will interact with promoters or enhancers leading to transcriptional gene silencing (TGS) or transcriptional gene activation (TGA). The putative miRNA recognition elements (MREs) could be recognized by miRNAs that mediate chromatin silencing complex assembly or de novo DNA methylation at the promoter region resulting in compact, silent heterochromatin and TGS. Unlike, when miRISC interacts with TATA-box motifs enhancing promoter activities leading to TGA through enrichment of chromatin-remodeling factors and active chromatin marks. Moreover, miRNAs interaction with enhancers result in TGA through chromatin remodeling and the enrichment of active marks at enhancer regions. NLS—nuclear localization signal sequence.

On the one hand, TNRC6A protein, also known as GW182, can be independently transported into the nucleus binding directly to Impβ and interacting with Impα [23,24]. On the other hand, TNRC6A facilitates the shuttling of miRNA containing AGO-2 into the nucleus via its own nuclear localization signal sequence [25]. Besides, AGO-2 can be imported into the nucleus via IPO8 only when it loads mature miRNA [26]. Figure 1 illustrates the different ways of delivering RISC proteins and miRNAs from the cytoplasm into the nucleus.

Moreover, it is proposed that miRNA nuclear localization can also be controlled by nuclear localization signal sequences in miRNA molecules or full processing of pre-miRNAs in the nucleus. Several studies show that various motifs, including AGUGUU-motif, 5′-UUGCAUAGU-3′ and 5′-AGGUUGKSUG-3′ motifs (where K is a uridine or a guanine) as well as the consensus ASUS sequence (where S is a cytosine or a guanidine) are presented in many miRNAs and are engaged in the nuclear translocation [27,28,29]. It is supposed that miRNAs translocation is controlled by RNA-binding proteins (RBPs), however, molecular pathways are now recognized. Regarding processing of pre-miRNA molecules and their loading into nuclear RISC complex, there are many uncertainties that need to be investigated.

Although the functions of nuclear miRNAs have not been fully elucidated, it is suggested that they can regulate both transcriptional rates and post-transcriptional levels of mRNAs. miRNA-promoter interaction mediated by AGO proteins may either suppress or activate transcription depending on the location of their target region and epigenetic status of the promoter [22,30]. Genome-wide analysis revealed that human promoters contain miRNA-seed matching sites, suggesting that miRNA-mediated transcription regulation is likely to be a common phenomenon [31]. On the one hand, Benhamed et al. demonstrated that AGO-2 and *let-7f* are involved in the transcriptional repression of proliferation-promoting genes regulated by the retinoblastoma (Rb)/E2F repressor complex in senescence [32]. The putative MREs for the *let-7f* have been localized in the promoters of two E2F-target genes *CDC2* and *CDCA8*. Similarly, nuclear *miR-522* suppresses transcription of *CYP2E1* gene by interacting with its promoter forming a DNA:RNA hybrid which probably prevents binding of Pol II and transcription factor [33]. On the other hand, Zhang et al. revealed that several miRNAs, such as *let-7i*, *miR-138*, *miR-92a* and *miR-181d* bind to the TATA-box motifs and enhance the promoter activities of interleukin-2, insulin, calcitonin or c-Myc, respectively [34]. Also, Cyclin B gene has a sequence located in its promoter that interacts with *miR-744-5p* and *miR-466d-3p* leading to transcriptional upregulation [35]. A recent study has revealed, that miRNAs (*miR-26a-1*, *miR-339*, *miR-3179*, *miR-24-1* and *miR-24-2*) are able to induce expression of neighboring genes and function as enhancer (*cis*-acting DNA elements) regulators [36]. Moreover, this study has also shown that *miR-24-1* (located in the enhancer region) increases expression of *FBP1* and *FANCC* genes and triggers direct chromatin state alteration of the *FBP1* enhancer that activate transcription. Another notable fact is that transcriptional gene silencing (TGS) and transcriptional gene activation (TGA) can be achieved by miRNA-mediated epigenetic regulation. Indeed, miRNA directs the RNA-induced transcriptional silencing complex (RITS), which consists of chromatin remodeling enzymes (e.g., HDAC1, EHMT2 and EZH2) and DNA methyltransferase (DNMT3A), to promoter leading to the transition of active chromatin structure to silent heterochromatin [31]. According to the study carried out by Kim and co-workers *miR-320* directs to the promoter region AGO-1 that acts as the effector protein for transcriptional silencing of *POLR3D* gene [37]. Furthermore, simultaneous enrichment of tri-methyl histone H3 lysine 27 (H3K27me3, a repressive chromatin mark) and EZH2, a histone methyltransferase that mediates H3K27me3, has been observed at the *POLR3D* promoter [37]. Another study has revealed that *miR17-5p* and *miR20a*, encoded within a poly-miRNA cluster *miR-17-92*, are involved in the acquisition of heterochromatin marks at the promoters through seed-paring manner [38]. miRNA-mediated TGS is involved in cell differentiation processes. For example, during granulopoiesis *miR-223*-RISC interaction with the promoter of nuclear factor I-A (*NFI-A*) results in the recruitment of Polycomb group complex and histone-modifying enzymes that repress transcription of *NFI-A*, an important step for granulocytic differentiation [39]. It is postulated that specific miRNA can initiate TGS through de novo DNA methylation or chromatin modification in human cancer cells. In fact, *miR-10a* with AGO-1 and AGO-3 reduces *HOX4* expression in human breast cells mediating in de novo DNA methylation and accumulation of repressive chromatin marks (H3K27me3 and H3K9me2, di-methyl histone H3 lysine 9) at its promoter [40].

In contrast, AGO-miRNA complex may activate the expression of target loci by either disruption of the recruitment of silencing proteins (e.g., PRC2) to lncRNAs (long non-coding RNAs) or recruitment of protein complex containing transcriptional activators (e.g., transcription factors) [31,41]. In the nucleus, lncRNAs regulate epigenetic silencing of adjacent genes through recruiting chromatin-remodeling factors in close proximity of their promoters [42]. In case of *miR-744* and *Ccnb1* gene, miRNA-mediated TGA rely on the recruitment of AGO proteins and RNA Pol II enrichment as well as active chromatin marks (such as H3K4me3, tri-methyl histone H3 lysine 4) at the regulated gene promoters [35]. Moreover, *miR-373* activates transcription of E-cadherin and *CSDC2* genes only via enrichment of RNA Pol II at their promoters [43], while *miR-205* induces the expression of *IL24* and *IL32* tumor suppressor genes by targeting specific sites in their promoters as well enrichment of RNA Pol II and active chromatin modifications [44].

Similar to cytoplasmic miRNAs, nuclear miRNAs can also mediate post-transcriptional gene silencing (PTGS) inducing degradation of target mRNAs. Several studies suggest that miRNAs contribute to the regulation of miRNA precursors and lncRNA transcripts [31]. For instance, mouse nuclear *miR-709* is involved in the post-transcriptional regulation of the pri-*miR-15a/miR-16-1*, binding to a 19-nt recognition element and preventing processing of primary transcripts, thus, nuclear miRNAs can influence the biogenesis of other miRNAs suggesting hierarchical structures among miRNAs [45]. Furthermore, some nuclear-retained lncRNAs are also regulated by AGO-miRNA complexes that interact with miRNA-complementary sequences located in lncRNAs, thus impairing their stability and function [42]. Indeed, the highly abundant lncRNA, metastasis associated lung adenocarcinoma transcript 1 (*MALAT1*), has two MRE’s which are recognized and bound by *miR-9* [46]. Subsequently, putative *miR-675-5p* binding site within *H19* RNA transcripts has been identified and the overexpression of *miR-675-5p* significantly downregulated the level of the *H19* transcript [47]. So far, several other non-coding RNAs directly targeted by miRNAs have been identified. Interestingly, a long non-protein coding RNA involved in mammalian X-chromosome inactivation, X (inactive)-specific transcript (*XIST)*, has seed-paring sites for *miR-210* which modulates its RNA level [48]. Additionally, *miR-671* directs AGO2-mediated cleavage of a circular antisense transcript of the *CDR1* gene and negatively regulates this non-coding antisense transcript [49].

### Regulation of Alternative Splicing

miRNAs are able to indirectly modulate alternative splicing by regulating translation of various splicing factors. However, mounting evidence suggests that AGO-miRNA complexes can affect the regulation of alternative splicing directly in the nucleus by epigenetic and non-epigenetic mechanisms. A co-immunoprecipitation study has identified multiple AGO-associated splicing factors, moreover, AGO-1, AGO-2 and DICER1 knockdown and overexpression experiments confirmed their involvement in splicing decisions at alternatively spliced exons [50,51]. Advanced molecular analyses were able to identify miRNA binding sites within intronic sequences in mouse and human brain as well as in human myocardial cells [12,52,53]. It is proposed that miRNAs-mediated compaction of chromatin structure at specific exon-intron junctions slows the rate of RNA *Pol II* elongation, which favors exon inclusion [54]. Surprisingly, exon skipping can be achieved by single-stranded oligonucleotides (ss-siRNA), ss-siRNA is incorporated by AGO-2 in the cytoplasm, then is transported into the nucleus where AGO2-ss-siRNA complex binds to the target mRNA and disrupts association with the splicing machinery [55].

Taken together, the above considerations illustrate the complex regulatory mechanisms of miRNA-mediated gene expression in the cytoplasm and the nucleus. It should be emphasized, that miRNAs are involved in many crucial cellular regulatory processes and may activate or inhibit gene expression at both transcriptional and post-transcriptional level. Thus, deregulation of miRNAs biogenesis and function can disrupt these processes and finally lead to a wide range of human diseases. Hence, miRNAs are valuable as diagnostic and prognostic biomarkers for many diseases, including cancer, diabetes mellitus, cardiovascular pathologies and neurological disorders. Moreover, miRNAs are considered as molecular targets of novel therapies and treatment strategies.

## 4. miRNAs As Potential Cancer Epi-Therapeutics

Over the past few decades growing evidence has linked epigenetic mechanisms with the regulation of gene expression. Epigenetic markers such as DNA methylation and post-translational modifications of histone tails can rearrange the structure of chromatin leading either to activation or repression of transcription activity (for details see reviews [56,57]). It is interesting that not only nucleotide sequences determine the level of gene expression but also epigenetic modifications are involved in this process. Epigenetic processes are orchestrated by multiple proteins (e.g., DNA methyltransferases, DNA demethylases and histone modifying enzymes), non-coding RNAs (e.g., miRNAs and lncRNAs) and environmental factors. Typically, loss of DNA methylation (hypomethylation) turns on gene transcription by altering the structure of chromatin. In turn, too much DNA methylation (hypermethylation) induces chromatin compaction and hinders the expression of genes. Therefore, disruption of epigenetic regulation can lead to inappropriate gene expression that impairs crucial biological processes resulting in the development of “epigenetic diseases”. The first “epigenetic disease” was cancer and it was established that patients with colorectal cancer had less DNA methylation levels in cancer tissues than from their normal tissue [58]. Growing evidence suggests that epigenetic changes, unlike DNA sequence mutations, are reversible, so it seems that these changes can be an ideal target for epigenetic treatments.

**Figure 2:**
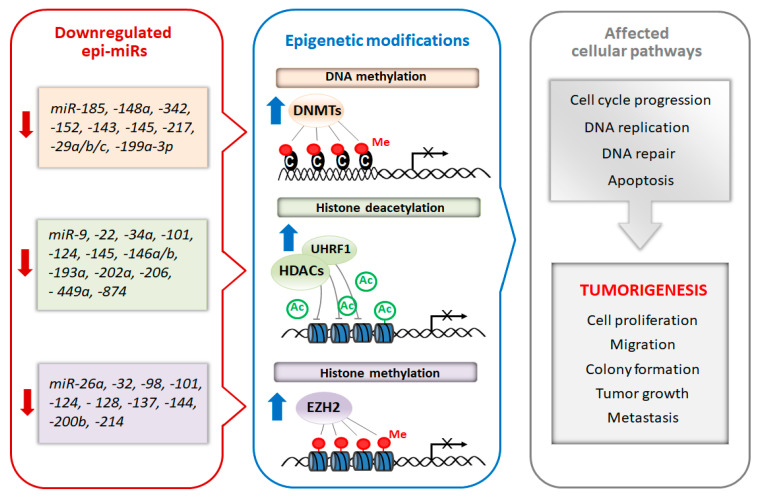
Schematic diagram of epi-miRs involved in the regulation of epigenetic modifiers and tumorigenesis. Suppression of miRNAs that regulate chromatin-remodeling enzymes lead to their overexpression. In turn epigenetic dysregulation resulting in improper regulation of genes responsible for different cell processes including cell proliferation, DNA repair and apoptosis thus triggering tumorigenesis.

Recently, a subclass of miRNAs, referred to as epi-miRNAs, that influence the expression of genes encoding epigenetic effector and reader proteins, has been identified [59]. Due to the important role of epi-miRs in the modulation of the epigenome, they are currently considered as potential therapeutic targets, especially in cancer. Manipulation of epi-miRs can affect the expression of epigenetically-regulated genes, such as oncogenes and/or tumor suppressor genes, involved in important cellular pathways including DNA replication, cell cycle progression and apoptosis [60,61]. The two types of miRs, oncomiRs and tumor-suppressor miRs, can be distinguished regarding their role in carcinogenesis. Generally, oncomiRs are up-regulated thereby increasing cancer cell proliferation and metastasis, in contrast the expression of tumor-suppressor miRs are down-regulated leading to enhanced tumorigenesis [62]. In this review, we focus on the therapeutic potential of tumor-suppressor epi-miRs that are downregulated in various types of cancer (casi el tinc. Emerging studies found that the decreased levels of epi-miRs promote cell proliferation, colony formation, tumor growth and metastasis [62,63,64]. Moreover, the suppression of some epi-miRs are responsible for the drug resistance of cancer cells [64,65]. Schematic relationship between downregulated tumor-suppressor epi-miRs, chromatin-modifying enzymes and cellular processes is shown in Figure 2.

To date, several causes have been found that influence the activity of miRNAs, their down-regulation is coupled with epigenetic silencing or genomic abnormalities, such as gene amplification, deletions and microdeletions (e.g., at *miR-101-1* loci) as well as mutations and chromosomal rearrangements [66,67]. Considering, the drug resistance of cancer chemotherapy (i.e., doxorubicin, cisplatin, paclitaxel), which are related to down-regulation of epi-miRs, their enforced expression appears to be an interesting approach to restore drug sensitivity (Table 1). Fabbri and co-workers revealed that the *miR-29* family (*29a*, *-b*, and *-c*) act as tumor suppressor miRs in lung cancer and regulate transcript levels of *DNMT3A* and *DNMT3B* [68]. Moreover, it has been established that synthetic epi-miR, *miR-29b* oligonucleotides, potentiates a hypomethylating effect of DNMT1 inhibitors (decitabine or azacitidine) resulting in better AML response for treatment probably due to the inhibition of other DNMT isoforms that are not efficiently suppressed by these agents [69]. Another study showed that synthetic *miR-29b* mimics inhibit *HDAC4* expression in multiple myeloma cell lines, reduce migration potential and increase apoptosis, therefore, this approach could offer a novel targeted therapy [70]. In addition, a recent study has shown that *miR-148a* combination therapy with either cisplatin or doxorubicin significantly enhanced apoptosis in urothelial cell carcinoma of the bladder cell lines [71]. Importantly, cancer stem cells (CSCs) are characterized by their ability to self-renew and resistance to standard chemotherapy, during remission can regenerate a tumor identical to the original one. An elegant study by Iliopoulos and colleagues uncovered that the combinatorial therapy of doxorubicin with epi-miR (*miR-200b*) was more effective than doxorubicin alone, blocking tumor growth and preventing relapse [72]. Interestingly, many natural agents, such as resveratrol, curcumin and glabridin used for epigenetic therapy, among others, exert their potent anti-tumor effects by enhancing expression of epi miRs. For instance, resveratrol causes up-regulation of *miR-137* in neuroblastoma tumors [73], curcumin increases levels of *miR-29a* and *miR-185* in hepatocellular cancer cells [74], in turn glabridin potentiates expression of *miR-148a* in breast cancer cells [75].

Epi-miRs-targeted cancer therapy seems to be a promising approach since it is able to influence not only a single gene, but multiple pathways. It is possible to re-establish expression of epi-miRs by delivering synthetic miR mimics (double stranded RNA oligonucleotides directly loaded into RISC) or chemically modified poly(nucleic acids), however, cellular uptake of free synthetic miRs are limited because of the ease in which they a degraded in biofluids [76]. In order to overcome poor in vivo stability and improve efficient and specific-site delivery of miRs to the tumor, innovative delivery systems are required. Currently, both viral and non-viral systems are used to increase stability of miRNA oligonucleotides and enhance their therapeutic effect. Administration of epi-miRs via viral vectors (e.g., adenoviruses, adeno-associated viruses (AAV) or lentiviruses) is very effective, as shown by systemic intravenous injection of epi-miR, *miR-26a*, packaged into AAV vector, which inhibited progression of hepatocellular carcinoma in a mouse model [77]. However, due to the viral vectors possible toxicity and immunogenicity their use in clinical practice is limited. In this context, non-viral systems seem to be more promising, because of the control of their molecular composition, ease in manufacturing and relatively low immunogenicity. Different delivery systems including, lipid-based delivery system, synthetic polymers (e.g, polyethyleneimine (PEI)) and naturally occurring polymers (e.g., chitosan, protamine and atelocollagen) are applied to protect miRs from degradation (for details see the review [78]). For example, a novel transferrin-conjugated nanoparticle delivery system for synthetic epi-miR, *miR-29b*, was injected intravenously and significantly prolonged leukemic mice survival [79]. Despite significant advances made in delivery systems of miRs, substantial improvements will be necessary for achieving site-specific delivery.

The discovery of therapeutic epi-miRs potential in cancer therapy makes them attractive candidates for next-generation cancer treatment. It therefore seems likely that profiling of miRs expression and then using appropriate epi-miR-based therapeutics may revolutionize cancer treatment by enabling the reversal of the epigenetic program of tumor cells to a more normal state.

However, we realize that turning epi-miR-based therapy into clinical practice faces challenges. Indeed, some clinical trials with miRNA drugs have not always produced satisfactory results. For example, the FDA (Food and Drug Administration) halted phase I clinical trial of *miR-34a* mimic (drug: MRX34) used in patients with different types of cancer. Double-stranded *miR-34a* was encapsulated into a liposome-formulated nanoparticle and administered intravenously [80]. Although, preclinical studies were promising, immune-related serious adverse events (SAEs) appeared during phase I. Due to SAEs this clinical trial was terminated and future phase II trials of MRX34 for melanoma were withdrawn. In contrast human trial of *miR-16* mimic (drug: MesomiR-1) exhibited hopeful results in patients with pleural mesothelioma and non-small cell lung cancer. Double-stranded *miR-16* was delivered by non-living bacterial minicells with a targeting moiety (i.e., an anti-EGFR bispecific antibody that recognizes EGFR-expressing cancer cells) [81]. This is a new targeted therapy known as TargomiRs. The successful completion of phase I trial confirmed safety and early signs of antitumor activity of TargomiRs so phase II of the trial is expected to begin soon.

**Table 1:** Epi-miRs as potential cancer therapeutics.

Epi-miRNA	mRNA Target	Cell Lines	Methods	Animal Study	Results	References
**Breast cancer**						
*miR-34a*	*HDAC1* *HDAC7*	MCF-7, MDA-MB-231, BT-20, T47-D, PC3, DU-145, LNCaP, NIH:OVCAR, SK-OV-3, HeLa and non-transformed mammary MCF-10A cells	*miR-34a* mimics; luciferase reporter assay (in MCF-7, PC3, and MDA-MB-231 cells)	ND	*miR-34a* expression negatively correlates with tumor grades; transfection of *miR-34a* mimic reduces cell survival and increases the cytotoxicity of chemotherapy drugs; re-expression of *miR-34a* inhibits the tumorigenic activity of cancer stem cells (CSCs).	[82]
*miR-101*	*EZH2*	SKBR3	*pre-miR-101*; luciferase reporter assay (in SKBr3 cells)	ND	*miR-101* overexpression in SKBr3 attenuates cell proliferation, migration and inhibits the invasive potential.	[83]
*miR-128*	*BMI1*	SK-3rd, MCF-7 and SKBR3	lentivirus vector *miR-128*; luciferase reporter assay (in SK-3rd and MCF-7 cells)	ND	Ectopic expression of *miR-128* decreases cell viability and increases apoptosis and DNA damage in the presence of doxorubicin; ectopic *miR-128* expression sensitizes BT-ICs (breast tumor–initiating cells) to doxorubicin enhancing the DNA damage and pro-apoptotic effects.	[84]
*miR-148a*	*SMAD2*	MDA-MB-231 and Hs-578T	*miR-148a* mimics; luciferase reporter assay (in MDA-MB-231 cells)	BALB/c nude mice; MDA-MB-231 cells were injected s.c.; glabridin (GLA) was administered intragastrically each day.	GLA enhances the expression of *miR-148a*; GLA-treated tumors have increased expression of *miR-148a* and decreased expressions of SMAD2.	[75]
*miR-185*	*DNMT1*	MDA-MB-231, MDA-MB-361, MDA-MB-435, MDA-MB-468, MCF-7, T47D, BT-474, BT-20 and BT-483; normal mammary epithelial cell lines (HBL-100, 184A1 and MCF-10A)	*miR-185* mimics	Nude mice; MDA-MB-231 cells were injected s.c.; intratumoral injection of *miR-185* mimics.	Ectopic expression of *miR-185* inhibits cell proliferation and induces apoptosis; inhibits tumor growth in vivo.	[85]
*miR200b*	*SUZ12*	MCF-10A cells containing the ER-Src fusion gene, MCF7, SKBR3, MDAMB-231, MDA-MB-435, NSCCs (non-stem cancer cells)	*miR-200b*; luciferase reporter assay (in ER-Src cells)	Athymic nude mice; CSCs were pretreated with *miR-200b* and injected s.c.; ER-Src (treated and untreated with tamoxifen) were injected s.c. and then doxorubicin or combination doxorubicin and *miR-200b* was administered i.p.	*miR-200b* overexpression affects CSCs growth and reduces cell invasiveness; pretreatment of CSCs with *miR-200b* blocked tumor formation in vivo; combinatorial therapy (doxorubicin with *miR-200b*) causes regression of tumor growth and prevents relapse of the disease.	[72]
**Bladder cancer**						
*miR-101*	*EZH2*	T24, UM-UC-3 and TCCSUP	vector pcDNA3.1 with *pre-miR-101*; luciferase reporter assay (in UM-UC-3 cells)	ND	Restored *miR-101* expression inhibits cell proliferation, suppresses colony formation and hinders EZH2-mediated neoplastic progression.	[86]
*miR-124*	*UHRF1*	J82, T24, HEK 293 and SV-HUC-1	*miR-124* mimics; luciferase reporter assay (in HEK-293cells)	Male BALB/C-A mice; T24 cells were injected s.c. and then intratumoral injection was performed with miR-124 mimics.	*miR-124* overexpression attenuates cell proliferation, migration, invasion and vasculogenic mimicry; inhibits tumor growth in vivo.	[87]
*miR-144*	*EZH2*	T24	vector pcDNA–*miR-144*; luciferase reporter assay (in HEK293 cells)	ND	*miR-144* overexpression inhibits cell proliferation; decreases EZH2 protein levels.	[88]
*miR-145-5p* *miR-145-3p*	*UHRF1*	T24 and BOY	*pre-miR-145-5p* and *pre-miR-145-3p*; luciferase reporter assay (in T24 and BOY cells)	ND	Ectopic expression of either *miR-145-5p* or *miR-145-3p* suppresses cancer cell growth, migration and invasion and induces apoptosis.	[89]
*miR-148a*	*DNMT1*	SV-HUC-1, T24, TCCSUP, J82 and UM-UC-3	*miR-148a* mimics; cisplatin or doxorubicin treatment	ND	*miR-148a* overexpression reduces cell viability by promoting apoptosis; combinatorial therapy (*miR-148a*/cisplatin or *miR-148a*/doxorubicin) enhanced apoptosis.	[71]
**Colorectal cancer**						
*miR-9*	*UHRF1*	HCT116 and HT29	*miR-9* oligonucleotides; lentivirus vector *miR-9*; luciferase reporter assay (in HCT116 and HT29 cells)	ND	*miR-9* overexpression attenuates CRC cell proliferation and promotes cell apoptosis; reduces UHRF1 expression.	[90]
*miR-143*	*DNMT3A*	228, CaCO2, Clone A, HCT116, HT-29, MIP101 and SW480	*pre-miR-143*; luciferase reporter assay (in 228 and SW480 cells)	ND	Ectopic expression of *miR-143* inhibits cell growth, reduces clone formation; restored *miR-143* expression decreases tumor cell growth and soft-agar colony formation, and downregulates DNMT3A expression.	[91]
*miR-342*	*DNMT1*	SW480, HT29, HCT116 and HEK293T	*miR-342* oligonucleotides; lentivirus vector *miR-342*; luciferase reporter assay (in SW480 cells)	Female athymic BABL/c nude mice; cell lines stably expressing *miR-342* were injected s.c.	Enhanced *miR-342* expression inhibits cell proliferation and invasion; *miR-342* overexpression leads to demethylation and induction of tumor suppressor genes through blocking DNMT1 expression; *miR-342* overexpression inhibits tumor growth and lung metastasis in vivo.	[92]
**Endometrial cancer**						
*miR-101*	*EZH2*	SPAC-1-L and SPAC-1-S; HEC-50 and HOUA-I cell lines were derived from poorly-differentiated endometrioid EC (endometrial carcinoma)	vector with *pre-miR-101-3p*; luciferase reporter assay (in SPAC-1-L and HOUA-I cells)	ND	Ectopic overexpression of *miR-101* suppresses cell proliferation, attenuates the epithelial-mesenchymal transition associated cancer cell migration and invasion, abrogates the sphere-forming capacity and enhances chemosensitivity to paclitaxel.	[93]
**Esophageal cancer**						
*miR-203*	*BMI1*	EC9706 and KYSE150	lentivirus vector *miR-203*; luciferase reporter assay (in EC9706 cells)	Female nude mice and nonobese diabetic/severe combined immunodeficient mice; freshly prepared cells were injected s.c.	*miR-203* overexpression reduces colony formation, tumorigenicity ability and self-renewal of esophageal cancer stem-like cells; increases sensitivity to cisplatin.	[94]
**Gastric cancer**						
*miR-29b/c*	*DNMT3A*	AGS and BGC-823	miR-29b/c mimics; luciferase reporter assay (in BGC-823 cells)	ND	*miR-29b/c* overexpression decreases migration and reduces invasive ability; *miR-29b/c* suppresses the expression of DNMT3A.	[95]
*miR-146a* *miR-146b*	*UHRF1*	GC9811, GC9811-P, MKN28NM and MKN28M	*pre-miR-146a/b*; lentivirus vector *miR-146a/b*; luciferase reporter assay (in HEK293T and GC9811 cells)	Nude mice; metastasis assay: GC9811-P cells infected with miR-146a/b were injected into the tail vein.	Restored expression of *miR-146a/b* reduces the expression of UHRF1; upregulation of *miR-146a/b* suppresses metastasis.	[96]
*miR-148a*	*DNMT1*	SGC-7901, BGC-823 and GES-1 (human gastric epithelium-immortalized cell line)	*miR-148a* mimics	ND	*miR-148a* mimics suppresses cell proliferation; *miR-148a* overexpression decreases DNMT1 expression and induces the overexpression of MEG3 (lncRNA).	[97]
*miR-206*	*HDAC4*	SGC-7901, BGC-823, AGS, non-malignant gastric cell line GES-1 and HEK293T	*miR-206* mimics; vector with *miR-206*	Nude mice; SGC-7901 cells carrying P2GM-*miR-206* was injected s.c.	Ectopic expression of *miR-206* represses cell proliferation, colony formation, invasion and migration; *miR-206* promotes myogenic differentiation and blocks tumor growth in vivo.	[98]
**Glioblastoma**						
*miR-128*	*Bim-1*	U87MG, U251MG and U373MG	*pre-miR-128* mimics; lentivirus vector *pri-miR-128-1*; luciferase reporter assay (in U87, U251, and U373 cells)	Athymic mice; U87 cells stably expressing *miR-128* were implanted s.c.	*miR-128* expression reduces glioma cell proliferation, self-renewal in vitro and glioma xenograft growth in vivo.	[99]
*miR-128*	*SUZ12* *BMI1*	U87 malignant glioma (MG) and U251MG glioblastoma cells	*pre-miR-128*; lentivirus vector *miR-128*; luciferase reporter assay (in HEK293 cells)	Mut3 mice (hGFAP-cre; Nf1flox/+; Trp532/+).	*miR-128* overexpression reduces proliferative potential and colony formation; reestablishment of *miR-128* expression impairs glioma stem-like cells self-renewal and increases their radiosensitivity.	[100]
**Head and neck squamous cell carcinoma**						
*miR-874*	*HDAC1*	SAS, FaDu, HSC3, IMC-3, human fibroblast and MRC-5	mature *miR-874*; luciferase reporter assay (in SAS cells)	ND	Restoration of *miR-874* inhibits cell proliferation, induces cell cycle arrest and apoptosis.	[101]
**Hepatobiliary cancer**						
*miR-152* *miR-148a*	*DNMT1*	KMCH-1, Mz-ChA-1, TFK-1 and H69; Mz-IL-6 (KMCH-1 stably transfected with IL-6)	*pre-miR-152* and *pre-miR-148a*; luciferase reporter assay (in Mz-ChA-1 cells)	Male athymic nu/nu mice; Mz-IL-6 cells were injected s.c.	*pre-miR-148a* and *pre-miR-152* decreases DNMT-1 protein expression and reduces cell proliferation; *miR-148a* and *miR-152* expression was reduced in tumor cell xenografts in vivo.	[102]
**Hepatocellular carcinoma**						
*miR-22*	*HDAC4*	Hep3B and SMMC7721	*miR-22* mimics; luciferase reporter assay (in Hep3B cells)	Male BALB/c athymic nude mice; *miR-22* mimics transfected Hep3B or SMMC7721 cells were injected s.c.	Restoration of *miR-22* expression suppresses cell proliferation and endogenous expression of HDAC4 protein; *miR-22* transfection delays tumor formation and reduces tumor size in vivo.	[103]
*miR-29a* *miR-185*	*DNMT3A* *DNMT3B*	HepG2 and HuH-7	dendrosomal curcumin (DNC) treatment	ND	Overexpression of *miR-29a* and *miR-185* after dendrosomal curcumin (DNC) treatment, down-regulates the expression of DNMT1, 3A and 3B.	[74]
*miR-145*	*HDAC2*	Hep3B, HepG2, SNU-182, SNU-449 and PLC/PRF/5	*miR-145* mimics; vector with *miR-145*; luciferase reporter assay (in SNU-449 cells)	Male athymic nude mice; Hep3B cells transfected with *miR-145* were injected s.c.	Ectopic expression of *miR-145* inhibits cell growth and HDAC2 expression; inhibits tumor growth in vivo.	[104]
*miR-200a*	*HDAC4*	SMMC-7721 and HepG2	*miR-200a* mimics; lentivirus vector *miR-200a*; luciferase reporter assay (in SMMC-7721 cells)	Nude mice; HepG2 cells stably transfected with *miR-200a* were implanted s.c.	*miR-200a* inhibits cell proliferation and migration both in vivo and in vitro; *miR-200a* overexpression induces up-regulation of global acetyl-histone H3.	[105]
**Acute myeloid leukemia**						
*miR-29b*	*DNMT3A* *DNMT3B*	AML cell lines, Kasumi-1, MV4-11 and K562	*pre-miR-29b*; lentivirus vector *miR-29b*; luciferase reporter assays (in K562 cells)	ND	Enforced expression of *miR-29b* in AML cells reduces of the expression of DNMT1, DNMT3A, and DNMT3B; *pre-miR-29b* overexpression induces partial differentiation of AML blasts.	[79]
*miR-29b*	*DNMT3B*	primary AML blasts, K562 and Kasumi-1	synthetic *miR-29b*	Female nude mice; synthetic *miR-29b* oligonucleotides were injected directly into the tumors.	Restoring *miR-29b* expression, induces apoptosis and dampens cell growth in AML cells.	[106]
*miR-193a-3p*	*DNMT3A* *HDAC3*	HL60, U937, U937-A/E-HA, Kasumi-1, SKNO-1, SKNO-1-siA/E-RNA and KG1	*miR-193a* mimics; lentivirus vector *miR-193a*; luciferase reporter assay (in 293T cells)	Nude mice; SKNO-1 cells were injected s.c.; intratumor injection of *miR-193a*.	Enhanced *miR-193a* levels induce G1 arrest, apoptosis, and restores leukemic cell differentiation; decreases tumor size in vivo.	[107]
**Chronic myeloid leukemia**						
*miR-217*	*DNMT3A*	Bcr/Abl-expressing K562 cells	lentivirus vector *miR-217*; luciferase reporter assay (in K562DR cells)	Female immune deficient BALB/c nude mice; K562 cells were injected s.c.; drug administration: dasatinib or 5-AzadC or a combination of both dasatinib and 5-AzadC.	Forced expression of *miR-217* inhibits expression of DNMT3A and sensitizes cells to growth inhibition mediated by the tyrosine kinase inhibitors (prevents drug resistance).	[108]
**Multiple myeloma**						
*miR-29b*	*DNMT3A* *DNMT3B*	MM cell lines	*pre-miR-29b* mimics (formulated with a Neutral Lipid Emulsion (NLE) delivery system); lentivirus vector *miR-29b*; luciferase reporter assay (in INA-6 cells)	Male CB-17 severe combined immunodeficient (SCID) mice; MM cells were inoculated s.c.; *miR-29b* mimics were administered intratumorally and systemically via tail vein.	*miR-29b* mimics impair cell cycle progression and potentiate the growth-inhibitory effects induced by the demethylating agent 5-azacitidine; *miR-29b* mimics induce anti-tumor effects in vivo.	[70]
**Leukemia**						
*miR-143*	*DNMT3A*	AML (HL-60, NB4 and U937), CML (K562), acute erythroleukemia (HEL), T lymphocytic leukemia (Jurkat and CEM), B-cell lymphoma (CA46, Raji cells of Burkitt’s lymphoma) and multiple myeloma (U266)	lentivirus vector *miR-143*	ND	*miR-143* overexpression decreases DNMT3A mRNA and protein expression, reduces cell proliferation, colony formation and cell cycle progression as well as induces apoptosis.	[109]
**Lung cancer**						
*miR-29a*, *-b*, *-c*	*DNMT3A* *DNMT3B*	A549 and H1299	*pre-miR-29a*, *-29b-1*, *-29c* oligonucleotides; luciferase reporter assay (in A549 cells)	Female nude mice; A549 cells transfected with pre-*miR-29a*, *-29b*, or *-29c*, were injected s.c.	Enforced expression of *miR-29s* restores normal patterns of DNA methylation, induces re-expression of methylation-silenced tumor suppressor genes and inhibits tumorigenicity in vitro and in vivo.	[68]
*miR-193a-3p* *miR-193a-5p*	*UHRF1*	SPC-A-1, SPC-A-1sci, A549, H1299, LC-21, H358 and HEK-293T	*miR-193a-3p/5p* mimics; lentivirus vector *miR-193a-3p/5p*; luciferase reporter assay (in HEK293T cells)	BALB/C-nu/nu nude male mice; metastasis assays: SPC-A-1sci cells stably expressing the *miR-193a-3p/5p*-mimic were injected into the tail vein.	*miR-193a-3p/5p* overexpression inhibits cell proliferation, migration, invasion and epithelial–mesenchymal transition (EMT); lung metastasis formation in vivo.	[110]
**Lymphoma**						
*miR-26a*	*EZH2*	human BL cell lines; murine MYC-induced lymphoma cell lines	vectors with mature *miR-26a*; luciferase reporter assay (in HEK-293 cells)	ND	*miR-26a* overexpression reduces cell numbers and results in an anti-proliferative effect.	[111]
**Melanoma**						
*miR-200c*	*BMI1*	WM35, WM793, WM115A, M3523A, 1205Lu and 293T	lentivirus vector *miR-200c*; vector pEZX-*miR-200c*	Male athymic nu/nu mice; *miR-200c*–WM115A cells were injected s.c.	*miR-200c* overexpression decreases cell proliferation, colony formation and migratory capacity as well as drug resistance and increases sensitivity to various chemotherapeutic agents (including cisplatin); inhibits melanoma xenograft growth and metastasis in vivo.	[112]
**Neuroblastoma**						
*miR-137*	*EZH2*	Mouse Neuro-2a (N-2a); human SH-SY5Y	*miR-137* mimics; resveratrol (RSV) treatment; luciferase reporter assay (in HEK293 cells)	ND	*miR-137* expression is up-regulated after RSV treatment; *miR-137* inhibits EZH2 expression after RSV treatment; *miR-137* regulates the EZH2-mediated apoptosis after RSV treatment.	[73]
*miR-124*	*EZH2*	Neural Stem Cells (NSCs) and HEK293T	mature *miR-124*	ND	*miR-124* overexpression down-regulates expression of Ezh2 and up-regulates neuron-specific Ezh2 target genes; promotes neuronal differentiation.	[113]
*miR-137*	*KDM1A*	IMR-32, SHEP, SKN-BE and HEK-293	*pre-miR-137*; luciferase reporter assay (in SHEP and HEK293 cells)	ND	Re-expression of *miR-137* increases apoptosis, decreases cell viability and proliferation, induces neuronal differentiation; downregulates KDM1A.	[114]
*miR-152*	*DNMT1*	SK-N-BE, SH-SY5Y, SK-N-AS and Kelly	*pre-miR-152*; luciferase reporter assay (in Kelly cells)	ND	Ectopic upregulation of *miR-152* declines cell invasiveness and anchorage-independent cell growth, contributing to the differentiated phenotype.	[115]
**Oral squamous cell carcinoma**						
*miR-32*	*EZH2*	SCC-4, SCC-9, SCC-25 and Tca8113; normal oral keratinocyte cell line (hNOK)	mature *miR-32* mimics; luciferase reporter assay (in Tca8113 cells)	ND	*miR-32* overexpression reduces cell proliferation, migration and invasion, promotes cell apoptosis; *miR-32* down-regulates the expression of EZH2.	[116]
**Ovarian cancer**						
*miR-15a* *miR-16*	*BMI1*	OVCAR-5, OV-167, OV-202, CP-70, A2780 and OSE (ovarian surface epithelial cell)	*pre-miR-15a*, *pre-miR-16*; luciferase reporter assay (in OV-202 and CP-70 cells)	ND	*miR-15a* or *miR-16* overexpression decreases cell proliferation and clonal growth; downregulates BMI1 protein levels.	[117]
*miR-152* *miR-185*	*DNMT1*	SKOV3, A2780, A2780/DDP (cisplatin-resistant), A549 and HepG2	*miR-152* and *miR-185* mimics; luciferase reporter assay (in SKOV3/DDP cells)	CD-1/CD-1 nude mice; SKOV3/DDP cells transfected with miR-152 that were injected i.p.	*miR-152* or *miR-185* overexpression increases cisplatin sensitivity by inhibiting proliferation and promoting apoptosis; promotes sensitivity to cisplatin through targeting DNMT1 directly.	[118]
**Pancreatic cancer**						
*miR-148a* *miR152*	*DNMT1*	MIA PaCa-2 and AsPC-1	*pre-miR-148b*, *pre-miR-152*; luciferase reporter assay (in MIA PaCa-2 and AsPC-1 cells)	ND	*miR-148b* and miR-152 overexpression inhibits cell proliferation and induces apoptosis; decreases DNMT1 expression, returns DNA methylation to normal patterns and induces re-expression of tumor suppressor genes.	[119]
**Prostate cancer**						
*miR-101*	*EZH2*	DU145	*pre-miR-101*; luciferase reporter assay (in SKBr3 cells)	Male nude athymic BALB/c nu/nu mice, DU145 stable cells overexpressing *miR-101* were injected s.c.	*miR-101* overexpression attenuates cell proliferation, migration and invasive potential; reduces colony formation; reduces tumor growth in vivo.	[83]
*miR-145*	*DNMT3B*	PC3	*miR-145* mimics; luciferase reporter assay (in PC3 cells)	ND	*miR-145* overexpression downregulates the expression of DNMT3B; sensitizes prostate cancer cells to X-ray radiation.	[120]
*miR-449a*	*HDAC1*	PC-3, DU-145, BPH-1 and LNCaP	mature *miR-449a* mimics; a longer, dicer-dependent *pre-miR-449a*; luciferase reporter assays (in PC-3 cells)	ND	*miR-449* expression arrests cell cycle, apoptosis; regulates cell growth and viability in part by repressing the expression of HDAC-1.	[121]
**Renal cell carcinoma**						
*miR-101*	*UHRF1*	786-O and Caki-1	*pre-miR-101-3p*; luciferase reporter assay (in 786-O cells)	ND	Restoration of *miR-101* inhibits cell proliferation, migration and decreases invasion activity; suppresses UHRF1 expression.	[122]
**Rhabdomyosarcoma**						
*miR-29*	Yin Yang 1 (*YY1*)	C2C12 myoblasts, RH30 and RD2	*pre-miR-29*; lentivirus vector *miR-29*; luciferase reporter assays (in MB cells)	Athymic nu/nu female mice; RH30 cells were injected s.c.; intratumoral injection of lentivirus with *miR-29*.	*miR-29* overexpression reduces cell growth and increases levels of the differentiation markers; intratumoral addition of *miR-29* stimulates myogenic differentiation; inhibits tumor growth in vivo.	[123]
**Testicular cancer**						
*miR-199a-3p*	*DNMT3A* (especially *DNMT3A2*)	Ntera 2 (NT2)	*miR-199a-3p* mimics; luciferase reporter assay (in NT2 cells)	ND	*miR-199a-3p* overexpression restores the expression of tumor-suppressor genes by affecting DNA methylation of their promoter regions.	[124]
**Waldenström macroglobulinemia**						
*miRNA-9* *	*HDAC4* *HDAC5*	BCWM.1, WM-WSU, MEC-1 and RL	pre-miRNA-9 *	ND	Restoring *miRNA-9* * levels induces toxicity, apoptosis and autophagy; supports down-modulation of HDAC4 and HDAC5 and up-regulation of acetyl-histone-H3 and -H4	[125]

ND—no data; s.c.—subcutaneous; i.p.—intraperitoneal injection. * miR-9-3p.

## 5. Conclusions

Knowledge in the miRNA field is steadily increasing and recent information about the mechanisms of action, especially their involvement in epigenetic regulation has shed new light on cellular regulatory networks.

Interestingly, mature miRNAs are present in both the nucleus and the cytoplasm, therefore they can be involved in the regulation of transcription and translation processes. Nuclear miRNAs can influence gene expression via transcriptional activation or transcriptional gene silencing and shaping alternative splicing, Cytoplasmic miRNAs mainly mediated translation inhibition, however, some miRNAs are capable of activating translation of their target mRNA. A growing body of evidence suggests that miRNAs can act as regulators of the cell epigenome through translation inhibition of proteins engaged in epigenetic control and/or interaction with lncRNA. Considering the pervasive role of miRNAs in numerous biological processes, especially tumorigenesis, better understanding of their role in epigenetic regulation will aid the development of new therapeutic strategies.

Currently, miRNA-based treatment approaches for cancer, including tumor-suppressor epi-miRs, are tested in in vitro and in vivo experiments. Although results seem promising further studies will be needed to clarify the safety and effectiveness of epi-miR therapy in clinical practice. We strongly believe that re-introduction of tumor-suppressor epi-miRs will allow for more effective, personalized therapies in the near future.
